# ASTE1 frameshift mutation triggers the immune response in Epstein-Barr virus-associated gastric cancer

**DOI:** 10.1038/s41392-021-00771-5

**Published:** 2022-01-05

**Authors:** Binhao Huang, Qin Li, Qirong Geng, Jiawen Lao, Jing Guo, Shenglin Huang, Dazhi Xu

**Affiliations:** 1 Department of Gastric Surgery, Fudan University Shanghai Cancer Center, and Shanghai Key Laboratory of Medical Epigenetics, Institutes of Biomedical Sciences, Fudan University, Shanghai, China; 2 Department of Oncology, Shanghai Medical College, Fudan University, Shanghai, China; 3 Department of Medical Oncology, Fudan University Shanghai Cancer Center, Shanghai, China; 4 State Key Laboratory of Oncology in South China, Collaborative Innovation Center for Cancer Medicine, Guangzhou, China; 5 Department of Gastric Surgery, Sun Yat-sen University Cancer Center, Guangzhou, China

**Keywords:** Cancer microenvironment, Gastrointestinal cancer

**Dear Editor**,

Epstein-Barr virus-associated gastric cancer (EBVaGC) is a subtype of GC that is characterized by the presence of EBV in cancer cells.^1^ EBVaGC is a relatively rare malignancy, accounting for only 10% of GC cases. Compared to other types of GC, EBVaGC presents a distinct tumor microenvironment with ample immune infiltration and increased expression of immune response genes. This unique microenvironment is assumed to have a better response to immunotherapy. However, the efficacy of immunotherapy in EBVaGC is inconsistent.^2,3^ In some studies, the PR rates were hardly to exceed 30%. Therefore, to improve efficacy and achieve precise treatment under the background of low response rate, it is urgent to identify the specific subgroup of EBVaGC which could benefit from immune therapy.

In this study, whole-genome and transcriptome sequencing were performed for 50 patients with EBVaGC. All cases included in this study were confirmed to be EBER-ISH-positive (Supplementary Fig. S1a). All RNA-seq data passed the quality check and showed a balanced expression level (Supplementary Fig. S1b). Among 510 genes upregulated in EBVaGC (Supplementary Fig. S1c), we detected enrichment of the “viral carcinogenesis”, “cytokine−cytokine receptor interaction”, “cell cycle” and “cell adhesion molecules” KEGG pathways (Supplementary Fig. S1d). In the GO analysis, “DNA conformation change” was most significantly enriched in EBVaGC, followed by “negative regulation of immune system process” and “response to extracellular stimulus” (Supplementary Fig. S1e). Integrative clustering of 1000 immune-related genes based on expression identified two groups, immune-active and immune-inactive, with distinct characteristics (Supplementary Fig. S2a). To summarize the differentially expressed genes in the immune-active group, we performed gene set variation analysis (GSVA) using the expression matrix and computed enrichment scores based on the immune cell-related gene set. Unsupervised clustering of the enrichment level was mapped to clinicopathological information and the newly identified immune subtype classification (Fig. 1a). Notably, the immune-active group showed an enhanced immune response (Fig. 1b).

**Fig. 1:**
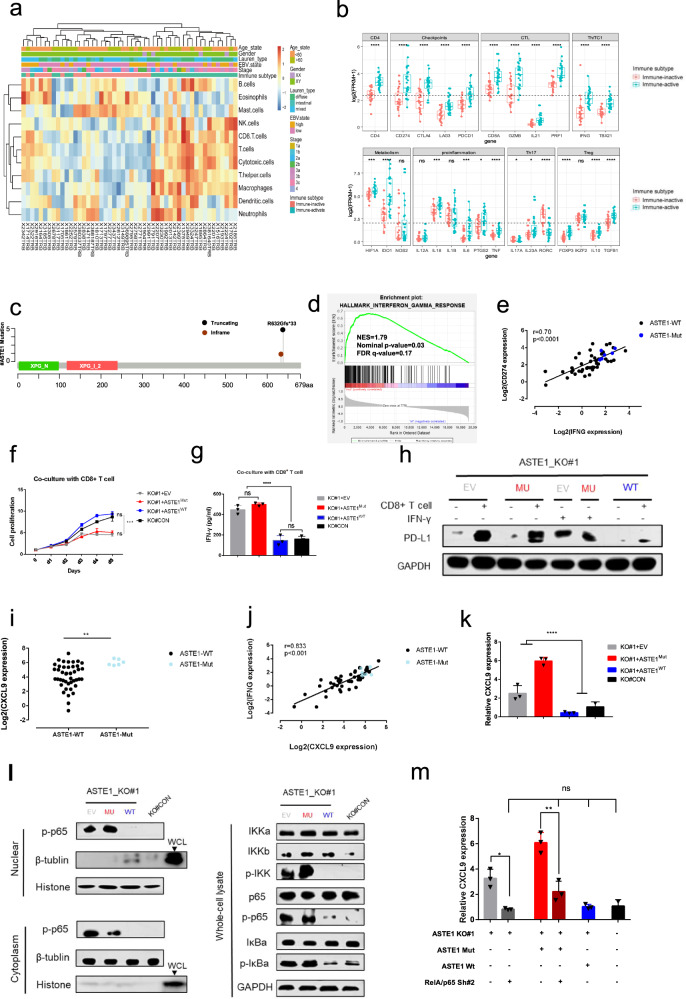
Immune classification and immune activation triggered by ASTE1 mutation in EBVaGC. **a** Gene set variation analysis (GSVA) based on immune cell-related gene set. Differential expressed genes are closely related to immune cells. **b** Expression characteristics of immune subtypes according to functional gene panel. Cluster 2 upregulated most of the immune genes and was named “immune-active” type; cluster 1 was named “immune-inactive” type. **c** Lollipop plots of mutant ASTE1 in sequenced EBVaGC samples. Somatic mutations in ASTE1 were indicated at the top of the corresponding domains in the protein. Among a total of six identified mutations, five had truncating mutations at the same site (R632Gfs*33). **d** GSEA revealed an enrichment of “IFN-γ response” pathway in ASTE1 mutation group. Normalized enrichment score (NES) = 1.79, Nominal *p*-value = 0.03, FDR *q*-value = 0.17. **e** Expression of CD274/PD-L1 was associated with IFNG (*r* = 0.70, *p* < 0.0001). **f** Cell proliferation was measured using the CCK-8 assay per co-culture day. **g** IFN-γ in the supernatant of the co-culture system was measured by ELISA. **h** PD-L1 expression of carcinoma cells after co-cultured with CD8^+^T cell or treated with IFN-γ **i**. ASTE1-mutant tumor upregulates CXCR3 ligand, CXCL9 (*p* < 0.01). The result was derived from RNA-seq of patient samples. **j** Expression of IFNG was associated with CXCL9 (*r* = 0.833, *p* < 0.001). **k** mRNA expression of CXCL9 in carcinoma cells was assessed by qPCR. **l** Left: results from western blot analyses of nuclear extracts and cytoplasm of transfected cells. Right: western blot analysis using anti-bodies against the respective phospho-sites (p-) or total protein in NF-κB pathway. **m** mRNA expression of CXCL9 was detected by qPCR after RNAi knockdown. Expression of CXCL9 reduced significantly in KO#1 + EV group and KO#1 + MU group

To evaluate the cellular compositions, the absolute abundance scores generated by MCP-counter were grouped by unsupervised clustering (Supplementary Figs. S2b, S2c). We obtained two subtypes with distinct cellular signatures. Based on composition between the groups, eight cell types exhibited a significant difference (Supplementary Fig. S2c). One group, which included 81.5% (22/27) of the immune-active patients, was characterized by an abundance of gamma delta T cells, follicular helper T cells, activated CD4 memory T cells, and M1-type macrophages. The other group, which included 91.3% (21/23) of the immune-inactive patients, exhibited enrichment of eosinophils, plasma cells, resting CD4 memory cells, and monocytes. The clustering results were in good accordance with the immune subtype results (Kappa = 0.721).

We also obtained RNA-seq data for 5 EBVaGC specimens from Kim’s study^3^ and evaluated the PD-L1 expression-related gene panel (Supplementary Fig. S2d). Interestingly, these patients presented an immune-active phenotype, with comparable PD-L1 expression and a similar gene expression pattern to our immune-active patients. This finding explained the result of 100% ORR for PD-1 inhibitors in these five patients. Furthermore, two patients in our study with obvious progression were evaluated after three courses of treatment with nivolumab, a PD-1 inhibitor (Supplementary Fig. S2e), and both patients exhibited immune-inactive behavior.

We further built a gene mutation panel including ASTE1, ARID1A, TNFAIP6, SMAD4A, and GIPC1 to predict the immune state (Supplementary Fig. S3a), and mutations in this panel predicted an immune-active phenotype (sensitivity = 80.8%, specificity = 65.2%, AUC = 0.73, kappa = 0.463) (Supplementary Figs. S3b, S3c). Notably, mutations in ASTE1 were identified in six patients (12%), whose immune states were all active. The frameshift mutation R632Gfs*33 was identified in 5 of 6 cases with mutation (Fig. 1c). Interestingly, a better prognosis was observed in the ASTE1 mutant group^4^ (Supplementary Figs. S3d, S3e).

As revealed by gene set enrichment analysis (GSEA) comparing ASTE1-Mut with ASTE1-WT, “IFN-γ response” was the set most significantly enriched among upregulated genes (Fig. 1d). The expression levels of IFNG and CD274 were upregulated in immune-active cancer and in Wang’s study^2^ (*p* < 0.001) (Supplementary Figs. S4a, S4b). Interestingly, mRNA expression of CD274 correlated positively with that of IFNG (*r* = 0.70, *p* < 0.0001) (Fig. 1e). Higher expression of PD-L1 was also validated at the protein level by immunohistochemistry (Supplementary Fig. S4c). Taken together, ASTE1-mutant cancer presents an active immune response, with a higher content of IFN-γ in the cancer microenvironment.

To explore the mechanism by which ASTE1 mutation activates the immune response, we knocked out the ASTE1 gene in the EBV^+^AGS cell line and selected KO#1 for transfection with ASTE1-MU (KO#1 + ASTE1^Mut^), -WT (KO#1 + ASTE1^WT^) and the empty vector (KO#1+EV) (Supplementary Fig. S5a). However, we did not observe any difference in cell proliferation or migration (Supplementary Figs. S5b, S5c). Interestingly, compared with the KO#CON group and KO#1+ ASTE1^WT^ group, cell growth decreased significantly when cocultured with activated CD8^+^ T cells (Fig. 1f and Supplementary Fig. S5d). Moreover, we detected higher levels of IFN-γ in the coculture supernatant of the KO#1+ ASTE1^Mut^ group and KO#1+EV group (Fig. 1g). In contrast, there was no change in the cell proliferation or immune response of CD8+ T cells when we overexpressed wild-type ASTE1 among EBV^+^AGS cells (Supplementary Figs. S5e, S5f). To validate the observed PD-L1 upregulation induced by IFN-γ, we compared PD-L1 protein expression in different genotypes. Notably, the KO#1+ ASTE1^Mut^ group and KO#1+EV group showed higher levels of PD-L1 protein after coculture or exogenous IFN-γ treatment (Fig. 1h).

Furthermore, ASTE1-Mut cancer presented higher expression levels of chemokines CXCL9, −10, and −11, ligands of CXCR3 (Fig. 1i, and Supplementary Figs. S6a, S6b). Also, expression of IFNG correlated with these chemokines (*r* = 0.833, 0.781, and 0.724 for CXCL9, −10 and −11, respectively, *p* < 0.001) (Fig. 1j and Supplementary Fig. S6c). In addition, qRT-PCR was performed to validate the increased CXCL9 and CXCL11 mRNA expression detected in the KO#1+ ASTE1^Mut^ and KO#1+EV cell lines (Fig. 1k and Supplementary Fig. S6d). However, there was no difference between the expression of IRF 1 and CXCL10 or STAT1 and STAT3 (Supplementary Fig. S7). We hypothesized that effectors regulating the transcriptional inflammatory response program were involved, and we thus assessed p-p65 by immunofluorescence (Supplementary Fig. S8a) and observed it mainly in, but not limited to, the nucleus in EBV^+^AGS cells (Fig. 1l). ASTE1-WT decreased p-p65 expression, while ASTE1-Mut had little effect. It strengthened the point that wild-type ASTE1 may suppress the inflammatory pathway. In addition, the expression level of p-p65 was relatively less in cytoplasm but more in the nuclear, indicating ASTE1-Mut might promote its nuclear translocation. Notably, expression of CXCL9 was significantly decreased in the KO#1+EV group and KO#1+MU group after RelA/p65 Sh#2 transfection (Fig. 1m and Supplementary Figs. S8b, S8c). These results indicated that ASTE1 mutation functions through activation of the NF-κB pathway in EBV^+^ GC cells.

ASTE1 mutation is reported to be associated with lymphocyte infiltration in MSI colorectal cancer.^5^ Here, we described for the first time the frameshift hotspot mutation R632Gfs*33 in ASTE1 (5/6) as being involved in EBVaGC. Strikingly, all EBVaGCs with ASTE1 mutations in our study were the immune-active type, which has been confirmed to predict an anti-PD1 response. Furthermore, ASTE1-mutant cancers show CXCL9-CXCR3 axis activation, which correlates with the efficacy of immunotherapy. Therefore, we propose a new mechanism by which ASTE1-mutant cancer cells autonomously express CXCL9 through NF-κB pathway activation, increase IFN-γ in the microenvironment and stimulate the immune response (Supplementary Fig. S9). Collectively, these findings strengthen the hypothesis that ASTE1 mutation has predictive potential for immunotherapy.

## Supplementary information

Supplementary Materials
