# Supplementary material for: ASTE1 frameshift mutation triggers the immune response in Epstein-Barr virus-associated gastric cancer
Source: Signal Transduct Target Ther. 2022 Jan 5;7:4. doi: 10.1038/s41392-021-00771-5 (PMC8727626; doi:10.1038/s41392-021-00771-5)
Supplement: Supplementary file 1 — Supplementary Materials [file 41392_2021_771_MOESM1_ESM.docx]

Supplementary Materials for

ASTE1 frameshift mutation triggers the immune response in Epstein-Barr virus-associated gastric cancer

Binhao Huang1,2†, Qin Li1,2†, Qirong Geng2,3, Jiawen Lao4,5, Jing Guo1,2*, Shenglin Huang1,2*, Dazhi Xu1,2*

Correspondence to: Jing Guo: [gjsysu@126.com](mailto:gjsysu@126.com); Shenglin huang: slhuang@fudan.edu.cn;Dazhi Xu: xudzh@shca.org.cn

**This PDF file includes:**

Materials and Methods

Figures. S1 to S9

Tables S1 to S4

Materials and Methods

**Patients and samples**

A total of 50 patients who underwent surgical resection at Sun Yat-sen University Cancer Center (SYSUCC), China, between 2014 and 2018 were included in this study. All patients were histologically diagnosed with gastric adenocarcinoma according to WHO classifications. EBV-encoded RNA (EBER)-in situ hybridization (EBER-ISH) and hematoxylin-eosin (HE) staining was performed on the serial section. Clinical and pathological data, including age, sex, tumor location, Lauren type and stage, were obtained from medical records and are summarized in Table S1. All study participants provided written informed consent for the use of their data for research. The study was approved by the institutional ethics committees of SYSUCC (Guangzhou, China). In addition, we obtained the RNA-seq data for 27 patients with EBVaGC from the TCGA database for validation and for 5 patients with EBVaGC from Kim’s study^1^ for comparison.

**Whole genome sequencing and transcriptome sequencing**

Genomic DNA was extracted from frozen tumor tissues and paired normal specimens using the QIAamp DNA Mini kit (QIAGEN, Valencia, CA). Total RNA was extracted using an RNeasy Mini Kit (QIAGEN, Valencia, CA). The library was prepared according to the manufacturer’s instructions and was subsequently subjected to high-throughput sequencing using a HiSeq X sequencer (Illumina HiSeq 2000 platform).

**Data processing**

WGS and RNA-seq data processing and quality checks were performed. High-quality reads were aligned to the NCBI Human Reference Genome hg19 using the default parameters of BWA-MEM. The Picard tool (http://broadinstitute.github.io/picard/) was used for postalignment processes, such as sorting, indexing, and tagging duplicates. The alignments were subjected to a local realignment and basic quality score recalibration (BQSR) around the INDEL using the Genome Analysis Toolkit (GATK) version 3.7 with default parameters^2^. Calling somatic substitutions (SNVs) and indels using MuTect2 as default parameters^3^. For all samples, the "LowEVS" variant in the Strelka output vcf file has been filtered out^4^. In the next steps, we used ANNOVAR to annotate all high confidence (HC) mutations. Web Gestalt evaluated pathways for significant mutations, with p-values <0.01^5^. RNA-seq FASTQ files were processed with the quality control tool FastQC (v0.11.5) (https://www.bioinformatics.babraham.ac.uk/projects/fastqc/) to evaluate the quality of sequencing reads. The raw data attained from sequencing were processed with Trimmomatic^6^. STAR 2-pass alignment (v2.5.3)^7^ was then performed to map those trimmed reads to corresponding reference genome, and StringTie^8^ was used to assemble transcripts. The Featurecounts^9^ tool was applied to aligned RNA-seq BAM files to count, for each gene, the number of aligned reads overlapped with its exons. The RNA-Seq gene expression raw read counts generated with Featurecounts were normalized into fragments per kilobase of transcript per million (FPKM) values using the following equation: $FPKM=\frac{{RC}_{g}*{10}^{9}}{{RC}_{pc}*L}$

where RCe is total exon fragments number, RCpc is the total fragments mapped to all genes, and L is the length of the gene (computed as sum of all exons in this gene).

**Differentially expressed genes (DEGs) and pathway analysis**

The FPKM expression matrix was then processed with the limma^10^ package to identify differentially expressed genes across paired tissues: tumor vs normal. Gene ontology (GO) analysis and Kyoto Encyclopedia of Genes and Genomes (KEGG) analysis were carried out with the R package “clusterProfiler”^11^ on differentially expressed genes between those three paired sets.

**Virus quantification**

A custom script was used for virus quantification. The transcripts per million (TPM) value of the viral transcript was obtained from the StringTie results. For all transcripts with the same viral infection, the maximum TPM value was utilized to represent viral mRNA expression.

**Immune signature clustering**

With the R package "ConsensusClusterPlus"^12^, we carried out hierarchical clustering with agglomerative average linkage and classified these patients into 2 clusters based on the 1000 most variable immune-related genes. These genes were derived from gene signatures proposed by Chung and immune-related genes with the highest frequency of C7 immune-related subunits in the Molecular Signatures Database (MSigDB)^13^. The newly identified clusters were used for further prognostic analysis. The R Bioconductor package “GSVA” (v.3.5) was applied with the default parameters to compute an enrichment score integrating the collective expression levels of 1000 most variable immune-related genes.

**Gene set enrichment analysis (GSEA)**

GSEA^14^ was performed using the GSEA program from the Broad Institute. The following options were selected or input into the software for analysis: (1) phenotypes analyzed, class1 and class2 produced by ConsensusClusterPlus; gene set database, c5.all.v6.1.symbols; number of permutations, 1000; permutation type, gene_set; significantly enriched gene sets, defined by a nominal P value of < 0.05. (2) phenotypes analyzed, ASTE1-Mut and ASTE1-WT; gene set database, halmarkers; number of permutations, 1000; permutation type, gene_set; significantly enriched gene sets, defined by a nominal P value of < 0.05

**Quantitative analysis of immune components**

The R package “MCP-counter”^15^ was applied to calculate the absolute abundance scores for 8 major immune cell types (CD3^+^ T cells, CD8^+^ T cells, cytotoxic lymphocytes, natural killer (NK) cells, B lymphocytes, monocytic lineage cells, myeloid dendritic cells, and neutrophils). xCell analysis^16^, a novel gene signature-based method, was further used to evaluate immune microenvironment with 64 immune and stromal cell types. The all three assessment used gene expression matrix of 50 EBVaGC patients as the input data. The result profiles were then hierarchically clustered and compared between two immune subtypes.

**Cell culture and reagents**

Human gastric cancer EBV^+^AGS cell line were obtained from the Dr. Musheng Zen (Sun Yat-sen University Cancer Center). The cell line was cultured according to standard protocols in DMEM medium (BasalMedia, L110). All the media were supplemented with 10% fetal bovine serum (Gibco,10, 270-106) and 1% penicillin-streptomycin (BasalMedia, S110B), and the cells were not passaged more than six times from collection to use.

**Expression vectors, plasmid transfection and lentiviral infection**

ASTE1 (NM_001288950) cDNAs were obtained from Genechem (Shanghai, China) and subcloned into the Ubi-MCS-3FLAG-CBh-gcGFP-IRES-puromycin vector (GV492) to generate Flag-gcGFP-ASTE1 expression vector. Frameshift mutation sequence (ASTE1 R657fs) were synthesized and subcloned into the same vector, verified by DNA sequencing.

Human p65 short hairpin RNAs (shRNAs) in Ubi-MCS-3FLAG-SV40-Neomycin vector (GV298) were purchased from Genechem. To generate stable cell lines expressing shRNAs, EBV+AGS cells were transfected with each lentivirus expression vector and packaging plasmid mix using Polyethyleneimine (PEI) DNA transfection reagent. The supernatant containing viruses was collected 48 h after transfection, filtered, and used for infecting target cells in the presence of 10 μg/ml of polybrene (Sigma-Aldrich, H9268) prior to drug selection with 1-2 μg/ml of puromycin for one week. Overexpression and knockdown (KD) efficiency were validated by immunoblotting after transfection.

LentiCas9-Blast (Addgene, #52962) and lentiGuide-Puro (Addgene, #52963) vectors were provided by Feng Zhang laboratory. The ASTE1 knock-out (KO) cell line was generated using the CRISPR/Cas9 system^17^ and was validated by Sanger sequencing and immunoblotting analysis. The individual sgRNA sequences are provided in Supplementary Table S2.

**CD8**^+^**T cell isolate and co-culture**

Peripheral blood mononuclear cells (PBMCs) were isolated from gastric cancer patients’ peripheral blood by percoll density-gradient centrifugation (Solarbio) and were treated with red blood cell lysate (Solarbio) based on standard protocol. To obtain CD8^+^ T cells, PBMC were stained with PE Mouse Anti-Human CD8 (BD, 555367) and sorted by flow cytometry. Activated by anti-CD3 (BD, 555336), anti-CD28 (BD, 555725) and IL-2 (BD, 5554562) for 2 days, CD8^+^ T cells were co-cultured with EBV^+^AGS cancer cells with 5:1 ratio.

**Cell viability assay**

The cells of interest (1×103 ~ 3×103 cells per well) were seeded into 96-well plates in triplicates overnight with 100 mL of complete growth medium, then treated with indicated drugs for the designed time. Cell viability was assayed using the cell counting kit-8 (CCK8) assay: add 10% CCK-8 solution to each well, incubate the plates in an incubator for 2 hours, and then measure the absorbance at 450 nm. The specific process refers to the manufacturer's protocol (Dojindo Molecular Technologies, Japan, CK04).

**ELISA assays**

Cell culture solution was centrifuged and take the supernatant for enzyme linked immunosorbent assay (ELISA). ELISA assays were run using IFN-γ ELISA kits (abs510007-96T) according to the manufacturer’s instructions.

**Real-time quantitative reverse transcription PCR (qRT-PCR)**

Total RNA was isolated from specimens or cells using RNeasy Plus Mini Kit (QIAGEN) following the manufacturer’s protocol. cDNA was synthesized using a PrimeScript RT Reagent Kit with gDNA Eraser (TAKARA, RR047A). Real-time qRT-PCR was carried out using SYBR Premix Ex Taq (TAKARA, RR420A) in triplicate on an ABI 7900HT Fast Real-time PCR system (Applied Biosystems). All of primers were synthesized by Sangon Biotech and the sequences of the primers are available in Supplementary Table S3. The results were analyzed with SDS v2.1 software and the 2−ΔΔCT method by normalization to the GAPDH levels.

**Western blot (WB) analysis**

cells were lysed in Pierce T-PER® Tissue Protein Extraction Reagent (Thermo Fisher Scientific Inc.) containing protease and phosphatase inhibitors (Bimake, B14001, B15001A+B). The lysates were centrifuged at 12000 rpm for 15 min, supernatants were collected, and protein concentrations were determined with a bicinchoninic (BCA) protein assay kit (Solarbio, Beijing, China, PC0020). A total of 20–30 μg of protein was separated by SDS-PAGE and transferred to PVDF membranes (Millipore, IPVH00010, ISEQ00010). The primary and secondary antibodies were described in Supplementary Table S4. ImageJ was used to quantify the immunoblotting results by protein band densities

**Immunohistochemistry (IHC) analysis**

For hematoxylin and eosin (H&E) staining, slides were stained with Mayer’s hematoxylin (Sigma-Aldrich) and 0.1% sodium bicarbonate and were counterstained with Eosin Y solution (Sigma-Aldrich). For IHC, the primary and peroxidase (HRP)-conjugated secondary antibody were listed in Supplementary Table S4. The positive-staining density was measured by a computerized imaging system mentioned above.

**Immunofluorescent staining**

Immunofluorescent staining was carried out as described previously^18^. Briefly, the adherent cells after drug treatment were washed in PBS, fixed in 4% paraformaldehyde, permeabilized in 0.1% Triton X-100, and blocked in 1% bovine serum albumin (BSA) in PBS. Cells were incubated with an anti-p-p65 (Abcam, ab194726,1:100) antibody in 1% BSA at 4◦C overnight, washed three times in PBS for five minutes each, and then incubated with the appropriate secondary antibody (Jackson ImmunoResearch,115-095-003/111-585-003). DNA staining was performed using ProLong™ Gold Antifade Mountant with DAPI (Invitrogen, P36931). Leica SP5 confocal laser scanning microscopy (Leica Microsystems, Buffalo Grove, USA) was used for immunofluorescence photography.

**Statistical analysis**

Potential associations between immune classification and clinical characteristics were assessed for significance using the χ2 test or Fisher’s exact test. Kaplan-Meier curves with log-rank tests was used to evaluate the survival outcomes. Intergroup differences in cell viability and gene expression were assessed for significance using the two-tailed Student’s t-test. In addition to the algorithms described above, all statistical analyses were performed in R version 3.4.1 software (R, CA, USA) and SPSS22.0 (SPSS Inc., Chicago, IL). A P value of <0.05 was considered statistically significant.

**Ethics approval**

The study was approved by the Institutional Ethics Committee of Sun Yat-sen University Cancer Center (YB2016-052).

**Data availability statement**

The data of this study are available from the corresponding authors upon reasonable request.

**References**

1.	Kim, S. T. et al. Comprehensive molecular characterization of clinical responses to PD-1 inhibition in metastatic gastric cancer. *Nat. Med.* **24**, 1449-1458 (2018).

2.	McKenna, A. et al. The Genome Analysis Toolkit: a MapReduce framework for analyzing next-generation DNA sequencing data. *Genome Res.* **20**, 1297-1303 (2010).

3.	Do, V. I. et al. Optimized pipeline of MuTect and GATK tools to improve the detection of somatic single nucleotide polymorphisms in whole-exome sequencing data. *BMC Bioinformatics*. **17**, 341 (2016).

4.	Wang, K., Li, M. & Hakonarson, H. ANNOVAR: functional annotation of genetic variants from high-throughput sequencing data. *Nucleic Acids Res.* **38**, e164 (2010).

5.	Lawrence, M. S. et al. Mutational heterogeneity in cancer and the search for new cancer-associated genes. *Nature*. **499**, 214-218 (2013).

6.	Bolger, A. M., Lohse, M. & Usadel, B. Trimmomatic: a flexible trimmer for Illumina sequence data. *Bioinformatics*. **30**, 2114-2120 (2014).

7.	Dobin, A. et al. STAR: ultrafast universal RNA-seq aligner. *Bioinformatics*. **29**, 15-21 (2013).

8.	Pertea, M. et al. StringTie enables improved reconstruction of a transcriptome from RNA-seq reads. *Nat. Biotechnol.* **33**, 290-295 (2015).

9.	Liao, Y., Smyth, G. K. & Shi, W. featureCounts: an efficient general purpose program for assigning sequence reads to genomic features. *Bioinformatics*. **30**, 923-930 (2014).

10.	Ritchie, M. E. et al. limma powers differential expression analyses for RNA-sequencing and microarray studies. *Nucleic Acids Res.* **43**, e47 (2015).

11.	Yu, G., Wang, L. G., Han, Y. & He, Q. Y. clusterProfiler: an R package for comparing biological themes among gene clusters. *Omics*. **16**, 284-287 (2012).

12.	Wilkerson, M. D. & Hayes, D. N. ConsensusClusterPlus: a class discovery tool with confidence assessments and item tracking. *Bioinformatics*. **26**, 1572-1573 (2010).

13.	Chung, W. et al. Single-cell RNA-seq enables comprehensive tumour and immune cell profiling in primary breast cancer. *Nat. Commun.* **8**, 15081 (2017).

14.	Subramanian, A. et al. Gene set enrichment analysis: a knowledge-based approach for interpreting genome-wide expression profiles. *Proc Natl Acad Sci U S A*. **102**, 15545-15550 (2005).

15.	Becht, E. et al. Estimating the population abundance of tissue-infiltrating immune and stromal cell populations using gene expression. *Genome Biol*. **17**, 218 (2016).

16.	Aran, D., Hu, Z. & Butte, A. J. xCell: digitally portraying the tissue cellular heterogeneity landscape. *Genome Biol*. **18**, 220 (2017).

17.	Ran, F. A. et al. Genome engineering using the CRISPR-Cas9 system. *Nat. Protoc.* **8**, 2281-2308 (2013).

18.	Sun, R. et al. FBXO22 Possesses Both Protumorigenic and Antimetastatic Roles in Breast Cancer Progression. *Cancer Res.* **78**, 5274-5286 (2018).

Figure. S1.

Fig. S1 Transcriptome profiling of EBVaGC specimens.

**a.** Diagnosis of EBVaGC. Hematoxylin-Eosin (HE) staining (left) and EBV-encoded RNA (EBER) in situ hybridization (right) at the same location for two continuous slice, magnification 200x. **b.** mRNA expression level for each specimen. **c.** Volcano map for differentially expressed genes associated with carcinoma, compared to normal tissue. Dashed line indicates a cut-off of |log Fold Change| >1, -log (p value) >2. **d.** GO signaling pathway enrichment in Biological Process (BP) set for differentially expressed genes. **e.** KEGG signaling pathway enrichment for differentially expressed genes.

Figure. S2.

Fig. S2 The immune cell composition and responses to PD1 inhibitor therapy.

**a.** Consensusclusterplus analysis based on immune genes. Two immune subtypes were obtained. **b.** Distribution map of cellular composition by MPC counter analysis for each specimen. **c.** Absolute abundance of infiltrating cells inferred by MCP-counter, with age, gender, Lauren type and EBV state on the top. **d.** Heatmap of gene set related to regulation of PD-L1 expression. EBVaGC in Kim’s study (NCT#02589496) shows a similar characteristic with immune active type. **e.** Computed tomography (CT) for size of metastasis lesion of patient X2771 before (left) and after (right) nivolumab treatment. Top: rectum; Middle: liver; Bottom: lung.

Figure. S3.

Fig. S3 Somatic mutations in ASTE1 and their prediction for a better prognosis.

**a.** Top driver mutations in EBVaGC. Each row represents one gene; each column represents one patient. Genes are listed with mutation frequency based on different mutation distribution in two groups. **b.** Predictive potential of mutation panel including ASTE1, ARID1A, TNFAIP6, SMAD4A and GIPC1. The standard for checking consistency was Cohen’s kappa value. With ASTE1 included, we tested all the combinations and finally selected ARIDA1, TNFAIP6, SMAD4A and GIPC1, which could maximize the kappa index. **c.** Mutations detected in this panel predicted an immune-active phenotype (Sensitivity=80.8%, Specificity=65.2%, AUC=0.73). **d,e.** Impact of mutations in the ASTE1 on overall survival and progression free survival of patients with GC. Data were derived from TCGA Pancancer Atlas database combined set and gastric cancer set respectively. These results were derived from online Web resource (http://cbioportal.org).

Figure. S4.

Fig. S4 Somatic mutations in ASTE1 indicated an active immune response.

**a.** Immune type is associated with tumor mutation burden (sensitivity=76.9%, specificity=65.2%). Cutoff point of exonic mutation count is 128.5. **b.** Immune-active group has more TMB-H patients than immune-inactive group (p=0.003). **c.** Higher mutation count and higher ratio of MSI-H subtype in ASTE1-Mut tumor. **d, e.** Comparison of IFNG and CD274 expression in immune-active, immune inactive and Kim’s study (NCT#02589496). *p<0.05, **p<0.01, ***p<0.001. **f.** Sections of formalin-fixed patient tumors carrying wild-type or mutant ASTE1 were stained against PD-L1. Left: wild-type, negative staining; right: mutant, positive staining.

Figure. S5.

Fig. S5 Cell proliferation and migration assay after transfecting with vectors.

**a.** ASTE1 knockout was confirmed by western blotting in EBV+AGS. KO#1 was selected for transfecting ASTE1-EV, -MU and –WT, respectively. **b.** Cell proliferation was measured using the CCK-8 assay. **c.** Carcinoma cell count (control) after cocultured with CD8+ T cell for 5 days. **d.** Wound healing assay of EBV+AGS after transfecting. **e, f.** Over expression of ASTE1 in EBV+AGS cell did not affect cell proliferation, migration or immune response of CD8+T cell.

Figure. S6.

Fig. S6 ASTE1 deficiency upregulated CXCL10 and CXCL11.

**a.** Heatmap of top 30 differentially expressed genes in “IFN-γ response”. ASTE1-mutant vs ASTE1-WT. **b.** ASTE1-mutant tumor upregulate CXCR3 ligand: CXCL10 and CXCL11. **c.** Expression of IFNG was associated with CXCL10, 11 (r=0.781 and 0.724 for CXCL10 and 11, respectively, p<0.001). **d.** mRNA expression of CXCL10 and CXCL11 in carcinoma cells was assessed by qPCR.

Figure. S7.

Fig. S7 mRNA expression of IRF1, STAT1 and STAT3 was measured by qPCR

Figure. S8.

Fig. S8 Immunofluorescence staining and knockdown of p65.

**a.** Immunofluorescence staining of p-p65 (red) in ASTE1-EV, -WT, or -MU transfected KO#1 cells and KO#CON cells. DAPI (blue) was used to identify nuclei. **b, c.** The RNAi knockdown efficiency was measured by western blotting in ASTE1-EV and –MU transfected KO#1 cells. shRelA/p65#2 was selected for subsequent analysis.

Figure. S9.

Fig. S9. Schematic view of the suggested mechanism

Table S1. Summary of clinical characteristics of patients with EBVaGC

| Characteristics | Count | Immune type (N=50) | |  | P value |
| --- | --- | --- | --- | --- | --- |
|  |  | Inactive (N=23) | Active (N=27) |  |  |
| Sex |  |  |  |  | 0.614 |
| Male | 46 | 22 (95.7%) | 24 (88.9%) |  |  |
| Female | 4 | 1 (4.3%) | 3 (11.1%) |  |  |
| Age |  |  |  |  | 0.057 |
| <60 | 17 | 11 (47.8%) | 6 (22.2%) |  |  |
| ≥60 | 33 | 12 (52.2%) | 21 (77.8%) |  |  |
| Tumor location |  |  |  |  | 0.784 |
| Upper | 14 | 4 (17.4%) | 10 (37.0%) |  |  |
| Middle | 26 | 13 (56.5%) | 13 (48.1%) |  |  |
| Lower | 10 | 6 (26.1%) | 4 (14.8%) |  |  |
| Tumor size (cm) |  |  |  |  | 0.088 |
| Median | 4.3 | 4.0 | 5.0 |  |  |
| Range | 1.5 - 13 | 1.5 - 13 | 1.5 - 13 |  |  |
| Lauren type |  |  |  |  | 0.670 |
| Intestinal | 9 | 5 (21.7%) | 4 (14.8%) |  |  |
| Diffuse | 24 | 6 (26.1%) | 18 (66.7%) |  |  |
| Mixed | 27 | 12 (52.2%) | 15 (55.6%) |  |  |
| Stage |  |  |  |  | 0.016 |
| I | 5 | 2 (8.7%) | 3 (11.1%) |  |  |
| II | 14 | 10 (43.5%) | 4 (14.8%) |  |  |
| III | 30 | 11 (47.8%) | 19 (70.4%) |  |  |
| IV | 1 | 0 (0.0%) | 1 (3.7%) |  |  |
| EBV state |  |  |  |  | 0.449 |
| High | 18 | 7 (30.4%) | 11 (40.7%) |  |  |
| Low | 32 | 16 (69.6%) | 16 (59.3%) |  |  |
| Data are presented as numbers and rates unless otherwise specified.  The chi-square test or Fisher’s exact test was used for categorical variables, while the Mann-Whitney U test was used for the tumor size. | | | | | |

Table S2. sgRNA sequences for Knocking out ASTE1

| **Primer name** | **Primer sequence (5' to 3')** |
| --- | --- |
| ASTE1-1F | CACCGGCTGGCATAGTCCGTGCACC |
| ASTE1-1R | AAACGGTGCACGGACTATGCCAGCC |
| ASTE1-2F | CACCGCCACATAGCACCGCAAAGAG |
| ASTE1-2R | AAACCTCTTTGCGGTGCTATGTGGC |
| ASTE1-3F | CACCGGGTGCTTACCGAGTTAGGTC |
| ASTE1-3R | AAACGACCTAACTCGGTAAGCACCC |
| ASTE1-4F | CACCGTTTACCAGAATGGGTATTAG |
| ASTE1-4R | AAACCTAATACCCATTCTGGTAAAC |
| ASTE1-5F | CACCGTATGAGGGAAACAACCGGTT |
| ASTE1-5R | AAACAACCGGTTGTTTCCCTCATAC |
| ASTE1-6F | CACCGATTGCCATAATCAACAGCCC |
| ASTE1-6R | AAACGGGCTGTTGATTATGGCAATC |

Table S3. Primers for qPCR

| **Primer name** | **Primer sequence (5' to 3')** |
| --- | --- |
| GAPDH_F | AAAGGGTCATCATCTCTG |
| GAPDH_R | GCTGTTGTCATACTTCTC |
| CXCL9_F | TGAGAAAGGGTCGCTGTTCC |
| CXCL9_R | GGGCTTGGGGCAAATTGTTT |
| CXCL10_F | TGCCATTCTGATTTGCTGCC |
| CXCL10_R | TGCAGGTACAGCGTACAGTT |
| CXCL11_F | GAGTGTGAAGGGCATGGCTA |
| CXCL11_R | ATGCAAAGACAGCGTCCTCT |
| STAT1_F | TGTATGCCATCCTCGAGAGC |
| STAT1_R | AGACATCCTGCCACCTTGTG |
| STAT2_F | CCGGGACATTCAGCCCTTTT |
| STAT2_R | CTCATGTTGCTGGCTCTCCA |
| STAT3_F | GAGCTGCACCTGATCACCTT |
| STAT3_R | CCCAGAAGGAGAAGCCCTTG |
| IRF1_F | GCCATTCACACAGGCCGATA |
| IRF1_R | GTGGAAGCATCCGGTACACT |

Table S4. Antibodies for western blot and immunohistochemistry

| **Antibodies names** | **Details** |
| --- | --- |
| ASTE1 | Novus Biologicals, NBP1-81661, 1:1000 |
| Flag | Cell Signaling Technology, CST-14793, 1:1000 |
| GAPDH | Cell Signaling Technology, CST-5174, 1:1000 |
| Histom H3 | Cell Signaling Technology, CST-4499S, 1:2000 |
| β-tublin | Abways, Q13509, 1:20000 |
| IKKa | Cell Signaling Technology, CST-11930, 1:1000 |
| IKKb | Cell Signaling Technology, CST-8943, 1:1000 |
| p-IKK | Cell Signaling Technology, CST-2697, 1:1000 |
| p65 | Cell Signaling Technology, CST-8242, 1:1000 |
| p-p65 | Abcam, ab194726, 1:1000 |
| IκBa | Cell Signaling Technology, CST-4814, 1:1000 |
| p-IκBa | Cell Signaling Technology, CST-2859, 1:1000 |
| PD-L1 | Cell Signaling Technology, CST-11930, 1:1000 |
| PD-L1 (immunohistochemistry) | Cell Signaling Technology, CST-11930, 1:200 |
| Anti-rabit IgG | Cell Signaling Technology, CST-7074, 1:1000 |
| Anti-mouse IgG | Cell Signaling Technology, CST-7076, 1:1000 |
